# Comparison of the perioperative outcomes of laparoscopic surgery, robotic surgery, open surgery, and transanal total mesorectal excision for rectal cancer: An overview of systematic reviews

**DOI:** 10.1002/ags3.12385

**Published:** 2020-08-29

**Authors:** Seiichiro Yamamoto

**Affiliations:** ^1^ Department of Gastroenterological Surgery Tokai University School of Medicine Kanagawa Japan

**Keywords:** laparoscopic surgery, open surgery, rectal cancer, robotic surgery, transanal total mesorectal excision

## Abstract

Regarding the surgical approaches for rectal cancer, many techniques have been reported in randomized controlled trials, meta‐analyses, and reviews of comparisons between two techniques, e.g. open surgery vs laparoscopic surgery, laparoscopic surgery vs robotic surgery, or laparoscopic surgery vs transanal total mesorectal excision. Since robotic surgery and transanal total mesorectal excision were developed after laparoscopic surgery had become an established minimally invasive technique, they have each been compared with laparoscopic surgery. Therefore, a review was performed to compare the surgical outcomes of robotic surgery and transanal total mesorectal excision, and to perform such comparisons among ≥3 of the above mentioned approaches, in the expectation that this review will serve as a reference for aiding treatment selection in future. The results of the current review suggest that all of the examined procedures have advantages and disadvantages, but that there are no decisive factors that could be used to select one procedure over any other. At the present time it cannot be demonstrated that laparoscopic surgery, robotic surgery, transanal total mesorectal excision, or open surgery is superior to the other techniques, and it is important to select the best technique for each patient from among those that a surgeon can perform. It is also important to maintain a flexible attitude that allows new techniques to be adopted as needed in the future.

## Graphical abstract

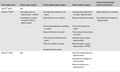

## 1 INTRODUCTION

Although open surgery (OpS) has conventionally been performed as the only form of radical surgery for rectal cancer, laparoscopic surgery (LaS) is widely indicated for rectal cancer as a minimally invasive surgery. The therapeutic outcomes of these procedures have been compared in several randomized controlled trials (RCTs) and meta‐analyses, which confirmed that there were no significant differences in long‐term prognosis.^1^, ^2^, ^3^, ^4^, ^5^, ^6^, ^7^, ^8^ In 2017, it was reported that the quality of LaS was significantly lower than that of OpS, and thus, concerns about the safety of LaS could not be refuted.^9^, ^10^, ^11^, ^12^ However, no data suggesting that the long‐term prognosis of LaS is worse than that of OpS were obtained in these clinical studies, and at present the safety of LaS is widely accepted when it is performed by a sufficiently experienced laparoscopic surgical team.^13^, ^14^, ^15^

On the other hand, the first robotic surgery (RoS) for rectal cancer was reported in 2006, and the frequency of RoS for rectal cancer has been increasing due to technical advances and the accumulation of experience among surgeons.^12^, ^16^, ^17^, ^18^, ^19^ In addition, the indications for and frequency of transanal total mesorectal excision (TaTME) have also been increasing, demonstrating its efficacy.^20^, ^21^, ^22^, ^23^, ^24^, ^25^ Important information regarding TaTME has been continuously reported from the international TaTME registry.^23^, ^24^, ^25^ However, concerns regarding its long‐term oncological outcomes are still reported, and TaTME remains a developing technique which should be performed with care.^12^, ^26^, ^27^ Recently, robotic TaTME, in which a robotic approach is used for the laparoscopic abdominal portion of TaTME, and the robotic transanal approach have been reported.^28^, ^29^, ^30^, ^31^, ^32^ At present, technologies continue to advance, and surgeons select the best approach from among the surgical techniques that they can perform based on their deep understanding of the merits and limitations of each approach.

Advances in surgical technology make it necessary to examine the efficacy of new technologies, and it is essential to examine the safety and efficacy of surgery for cancer, in addition to its long‐term prognosis. Regarding the four surgical approaches for rectal cancer, many comparisons between two techniques, e.g. OpS vs LaS, LaS vs RoS, and LaS vs TaTME, have been reported in RCTs, meta‐analyses, and reviews.^33^, ^34^, ^35^, ^36^, ^37^, ^38^, ^39^, ^40^, ^41^ These comparisons were performed between LaS and other approaches because LaS was the first type of minimally invasive surgery and was initially compared with conventional OpS, followed by RoS and TaTME, which were subsequently developed as different types of minimally invasive surgery. On the other hand, in actual clinical practice, few medical institutions or surgeons perform all four approaches, or even three of the approaches, on a routine basis, and thus it is difficult to conduct an RCT that compares three or four of the approaches at once.

This review was conducted to compare the surgical outcomes of RoS and TaTME, and to perform such comparisons among three or more approaches, in the expectation that it will serve as a reference for aiding treatment selection in the future. We reviewed studies that were published since 2018 in order to consider the latest findings.

## 2 ROBOTIC SURGERY VS TRANSANAL TOTAL MESORECTAL EXCISION

Since RoS and TaTME were developed after LaS had become established as a minimally invasive technique, RoS and TaTME have each been compared with LaS. In addition, since these techniques are indicated for the same patients, few medical institutions perform both RoS and TaTME. Thus, it is rare for RoS and TaTME to ever be compared directly.

Recently, some studies involving direct comparisons between RoS and TaTME have been published (Table 1). Perez et al^42^ compared the intraoperative and perioperative outcomes of 60 and 55 cases in which RoS and TaTME, respectively, were performed for low or middle third rectal cancer using data from a prospective database. In this study, all of the robotic surgical procedures were performed at one institution, and all TaTME procedures were conducted at another institution. The operating time and perioperative complications rates did not differ between the groups, and the circumferential resection margin (CRM) was wider in the RoS group than in the TaTME group, while none of the remaining oncological parameters exhibited intergroup differences. Therefore, it was concluded that both procedures should be considered equally feasible for low rectal cancer and as alternatives to conventional anterior resection (open or laparoscopic).

**Table 1:** Robotic surgery vs transanal total mesorectal excision

First author, year	Favors robotic surgery	Favors transanal total mesorectal excision
Perez,^42^ 2018	Circumferential margin	
	Distal margin	
Law,^43^ 2019		Operating time
		Intraoperative blood loss
		Abdominal incision
Gachabayov,^44^ 2019		

Law et al^43^ compared the intraoperative and perioperative outcomes of 80 cases of sphincter‐saving RoS and 40 cases of TaTME for rectal cancer by analyzing a prospective mono‐institutional database using propensity score matching. Some significant differences between baseline characteristics were observed including with regard to the level of the tumor from anal verge, and, after the matching procedure, the number of abdominal incisions and the size of the tumor were the only baseline characteristics that exhibited significant differences. The operating time was significantly shorter and the amount of intraoperative blood loss was lower in the TaTME group. Thus, they concluded that both RoS and TaTME can achieve favorable rectal cancer resection outcomes and that TaTME is associated with a shorter operating time, less intraoperative blood loss, and a higher rate of transanal specimen extraction.

Gachabayov et al^44^ compared histopathological metrics and/or complication rates between TaTME and RoS for lower, middle, or upper rectal cancer. They performed a systematic search and included six observational studies involving 1572 patients (TaTME: 811; robotic TME: 761) in their meta‐analysis. The CRM involvement rate, distal resection margin (mm), and complications rates did not differ between the procedures, and they concluded that compared with RoS performing TaTME for rectal cancer does not improve histopathological metrics or complication rates.

Although RoS and TaTME have various merits and demerits, both procedures can produce favorable intraoperative and perioperative rectal cancer resection outcomes when performed by a specialist.

## 3 OPEN SURGERY VS LAPAROSCOPIC SURGERY VS ROBOTIC SURGERY

Since 2018, two studies comparing OpS, LaS, and RoS have been published, which used different analytical methods (Table 2).

**Table 2:** Open surgery vs laparoscopic surgery vs robotic surgery

First author, year	Favors open surgery	Favors laparoscopic surgery	Favors robotic surgery
Zheng,^45^ 2020	Complete TME specimen (vs laparoscopic)		Retrieved lymph nodes (vs laparoscopic)
Kethman,^46^ 2020		Successful oncological resection (vs open, robotic)	Length of stay (vs open)
		Surgical site complications (vs open)	
		Readmission (vs robotic)	
		Length of stay (vs open)	

Abbreviation: TME, total mesorectal excision.

Zheng et al^45^ performed a systematic search of PubMed, Embase, the Cochrane Library, CNKI, and Web of Science to identify RCTs that compared any two of OpS, LaS, and RoS for lower, middle, or upper rectal cancer. Then, they conducted a network meta‐analysis with trial sequential analysis using a frequentist approach with random‐effects meta‐analysis, which included 22 RCTs. As a result, they found that OpS resulted in more complete TME specimens than LaS, but no significant differences were detected in the other comparisons. They also reported that, based on the P scores for the completeness of the TME specimen and CRM positivity, the best technique was OpS, followed by RoS and then LaS. However, this order was reversed when complications and mortality were considered. Therefore, they concluded that OpS might provide better pathological specimens and that minimally invasive techniques might have advantages in terms of lymph node harvesting, complications, and mortality.

On the other hand, Kethman et al^46^ reported contrasting results. They conducted a multicenter, quasi‐experimental cohort study, involving propensity score weighting, which included adult patients who underwent lower, middle, or upper rectal cancer resection at hospitals that were participating in the American College of Surgeons National Surgical Quality Improvement Program in 2016. Compared with LaS, OpS and RoS were associated with a decreased likelihood of successful oncological resection, and OpS was associated with an increased likelihood of surgical site complications and longer postoperative hospital stays.

Although many studies comparing LaS and RoS have been published, few studies have reported on three‐way comparisons that also involved the conventional method, OpS. Thus, further studies are needed to clarify the clinical outcomes of these procedures and factors that influence the choice of treatment.

## 4 OPEN SURGERY VS LAPAROSCOPIC SURGERY VS TRANSANAL TOTAL MESORECTAL EXCISION

Since 2018, two studies in which OpS, LaS, and TaTME were compared at single institutions have been published (Table 3).

**Table 3:** Open surgery vs laparoscopic surgery vs transanal total mesorectal excision

First author, year	Favors open surgery	Favors laparoscopic surgery	Favors transanal total mesorectal excision
Perdawood,^47^ 2020	Distal resection margin (vs laparoscopic, TaTME)	Retrieved lymph nodes (vs open)	Specimen quality (vs laparoscopic)
		Intraoperative blood loss (vs open)	Retrieved lymph nodes (vs open)
			Intraoperative blood loss (vs open, laparoscopic)
			Conversion to open procedure (vs laparoscopic)
			Operating time (vs open, laparoscopic)
			Anastomotic leakage (vs open)
			Hospital stay (vs open, laparoscopic)
			Readmission (vs laparoscopic)
Chen,^48^ 2020	Operation time (vs laparoscopic, TaTME)	Operation time (vs TaTME)	Intraoperative blood loss (vs open)
		Intraoperative blood loss (vs open)	Circumferential margin (vs open)
		2‐year disease‐free survival (vs open)	2‐y disease‐free survival (vs open)

Abbreviation: TaTME, transanal total mesorectal excision.

Perdawood et al^47^ conducted a case‐matched study, based on data from a prospectively maintained database of lower, middle, or upper rectal cancer patients who underwent TaTME, and a retrospective chart review of patients who underwent laparoscopic TME (LaTME) or open TME (OpTME) prior to the period covered by the database. The baseline characteristics of the three groups were comparable, and TaTME resulted in lower rates of incomplete TME specimens than LaTME, but not OpTME, and the other pathological results of TaTME were not significantly superior to those of LaTME or OpTME. On the other hand, while TaTME resulted in shorter operation times, less intraoperative blood loss, and shorter hospital stays, the complications and mortality rates of the three groups were comparable.

Chen et al^48^ compared the intraoperative and perioperative outcomes of 39 patients who underwent TaTME, 64 patients who underwent LaS, and 23 patients who underwent OpS for lower recal cancer. Regarding their baseline characteristics, the tumor location was lower in the TaTME group than in the other groups. TaTME resulted in a longer operation time than the other two groups; however, this can be explained by the fact that only one team performed TaTME. TaTME achieved better pathological results and disease‐free survival than OpS, but was not significantly superior to LaS. They also reported that there were no patients with CRMs of <1 mm in the TaTME group, whereas the equivalent frequencies for the LaS and OpS groups were 7.8% and 13.0%, respectively (*P* = .035). Moreover, the patients in the TaTME and LaS groups also significantly exhibited better disease‐free survival than those in the OpS group (*P* < .01).

Both studies were retrospective and single‐institutional, and further studies are needed to evaluate the short‐term surgical outcomes and long‐term oncological results of these approaches.

## 5 OPEN SURGERY VS LAPAROSCOPIC SURGERY VS ROBOTIC SURGERY VS TRANSANAL TOTAL MESORECTAL EXCISION

Comparisons of OpS, LaS, RoS, and TaTME have been performed using several methods (Table 4).

**Table 4:** Open surgery vs laparoscopic surgery vs transanal total mesorectal excision

First author, year	Favors open surgery	Favors laparoscopic surgery	Favors robotic surgery	Favors transanal total mesorectal excision
ESCP,^49^ 2017	NA			
Simillis,^50^ 2019	Operating time (vs laparoscopic, robotic)	Intraoperative blood loss (vs open)	Intraoperative blood loss (vs open, laparoscopic)	Operating time (vs robotic)
	Incomplete or nearly complete TME (vs laparoscopic)	Operating time (vs robotic)	Wound infection (vs open)	Circumferential margin (vs laparoscopic)
		Overall postoperative morbidity (vs open)	Time to first flatus (vs open)	
		Wound infection (vs open)	Time to bowel movement (vs open)	
		Time to bowel movement (vs open)	Hospital stay (vs open, laparoscopic)	
		Time to oral diet (vs open)	Distal margin (vs open, laparoscopic, TaTME)	
		Hospital stay (vs open)		
Rausa,^51^ 2019	NA		Overall complications (vs laparoscopic)	
			Anastomotic leakage (vs laparoscopic)	
			Wound infection (vs laparoscopic)	


Abbreviations: NA, not applicable; TaTME, transanal total mesorectal excisionTME, total mesorectal excision.

The 2017 European Society of Coloproctology (ESCP) collaborating group conducted a prospective, observational, multicenter study in accordance with a pre‐specified protocol, which included lower, middle, or upper rectal cancer patients who were scheduled to undergo elective total mesorectal excision for malignancy via any surgical approach.^49^ Interestingly, they included patients that were scheduled to undergo RoS in the abdominal region and the TaTME approach in the transanal region. Overall, 9.0% of patients suffered anastomotic leakage. In the univariate analyses, both TaTME and robotic TaTME (*P* = .02) were found to be associated with a higher risk of anastomotic leakage than LaS. However, this association was lost after controlling for patient and disease factors, while strong associations with low rectal anastomosis and male sex remained. The positive CRM rate varied between the operative approaches: LaS: 3.2%, TaTME: 3.8%, OpS: 4.7%, RoS: 1%. They concluded that the TaTME approach is widely performed and is associated with acceptable surgical and pathological results.

Simillis et al^50^ conducted a systematic literature review, involving a Bayesian network meta‐analysis, which compared OpS, LaS, RoS, and TaTME for lower, middle, or upper rectal cancer. The review included 29 RCTs. Intraoperative blood loss was lower in the RoS group than in the OpS and LaS groups. The operative time was significantly longer in the RoS group than in the other groups. LaS resulted in a lower overall postoperative morbidity rate and fewer wound infections compared with OpS. The time to defecation was longer after OpS than after LaS or RoS. The postoperative hospital stay was longer after OpS. LaS resulted in higher frequencies of incomplete or nearly complete mesorectal excision compared with OpS, and a higher positive CRM rate compared with TaTME. RoS produced longer distal resection margins than the other approaches. Finally, they concluded that the different techniques resulted in comparable perioperative morbidity and long‐term survival rates, and that LaS and RoS might improve postoperative recovery, whereas OpS and TaTME might improve oncological resection.

Rausa et al^51^ also conducted a systemic review of 23 studies, in which they used network meta‐analysis to compare LaS, RoS, and TaTME for lower, middle, or upper rectal cancer. They reported that RoS, TaTME, and LaS produced similar outcomes with respect to macroscopic mesorectal excision, lymph node harvesting, and radial margin involvement, which were reflected by comparable local and distant recurrence rates. Moreover, all three surgical approaches exhibited similar overall complication rates. Conversely, RoS was associated with a significantly lower risk of anastomotic leakage than LaS, although potential selection bias cannot be excluded. They concluded that all three surgical techniques were comparable in terms of TME quality and oncological outcomes and considered that good outcomes were achieved by individual surgeons selecting appropriate approaches based on their expertise.

## 6 DISCUSSION

Based on previous studies and this review, it is suggested that individual rectal cancer resection procedures have advantages and disadvantages, and that there are no decisive factors that could be used to select one procedure over any other. Since the superiority of LaS, RoS, or TaTME cannot be clearly demonstrated at this time, an appropriate procedure should be selected for each case based on the experiences of the medical institution and surgical team.

On the other hand, all of the examined studies suggested that the cost of RoS is a disadvantage.^52^, ^53^ However, the cost of RoS will decrease as the number of cases increases and a market for robot technology is created. Some studies have suggested that considering the positive effects of RoS on quality of life, the total medical costs of RoS and LaS are almost the same.^54^, ^55^ However, it is very unlikely that robots will be available for use in all medical institutions together with surgeons who have received sufficient education about RoS in the near future. On the other hand, since TaTME is most beneficial when it is performed in both the abdominal and perineum regions simultaneously, it is necessary to prepare two sets of laparoscopic surgical devices to be used by two experienced medical teams for each patient. However, two experienced medical teams using two sets of laparoscopic surgical tools could perform LaS in two patients. In this regard, LaS is superior with respect to manpower and medical costs.

In 2020, the world faces both medical and economic crises beyond our experience in the past 100 years due to the COVID‐19 pandemic. Surgeons cannot continue to perform surgery without considering the possibility of COVID‐19 infections, and it is estimated that COVID‐19 will have a large impact on future therapeutic policy and the selection of surgical techniques for rectal cancer. Under these social and medical circumstances, this review was performed with the expectation that it will serve as a reference for aiding treatment selection in the future, when the long‐term outcomes of RoS and TaTME in comparison with LaS will be better known. However, as mentioned above, LaS, RoS, and TaTME each have specific advantages and disadvantages. In addition, high‐quality surgery cannot be achieved using all of these procedures at all medical institutions. At present, there is no answer which procedure is superior to another procedure, and it is important that the surgeon selects the best technique for each patient from among those that they can perform. It is important to maintain a flexible attitude towards absorbing new techniques as needed in the future; however, it is also acceptable to only start learning a new procedure after its technical and oncological safety have been established since LaS, RoS, and TaTME each have specific advantages and disadvantages, and evaluations of these procedures are currently ongoing.

## DISCLOSURE

Funding Information: Declaration of prior publication.

Conflict of Interest: The authors declare that they have no conflicts of interest.
